# A tribute to Professor Robert Schoysman (1928 – 2016)

**Published:** 2016-09

**Authors:** W Ombelet, J Gerris, M Merckx

**Affiliations:** Editor “Facts, Views and Vision in ObGyn”, Genk Institute for Fertility Technology, Department of Obstetrics and Gynaecology, Ziekenhuis Oost Limburg, Schiepse Bos 6, 3600 Genk, Belgium; President Flemish Society of Obstetrics and Gynaecology, Women’s Clinic, University Hospital Ghent, De Pintelaan 185, 9000 Ghent, Belgium; Division of Pediatric Gynaecology, University Hospital St Pieter and Erasmus Brussels & ICRH Ghent Belgium

## Abstract

We honour the life of Robert Schoysman a pioneer and visionary in reproductive medicine, internationally renowned gynaecological surgeon and one of the pioneers of in vitro fertilization (IVF) in Belgium. From the early sixties he was interested in different options of male infertility treatment, including surgery and donor insemination. He was the pioneer of microsurgical epididymal sperm aspiration (MESA) and SUZI (subzonal sperm injection) in the early nineties. For those who were privileged to have worked with him he was a friend, a dedicated mentor and an enthusiastic teacher with vision and endless imagination.

“That what we have, we prize not to the worth, whilst we enjoy it, but being locked and lost, why, than we rack the value“

William Shakespeare - 1598

**Figure:**
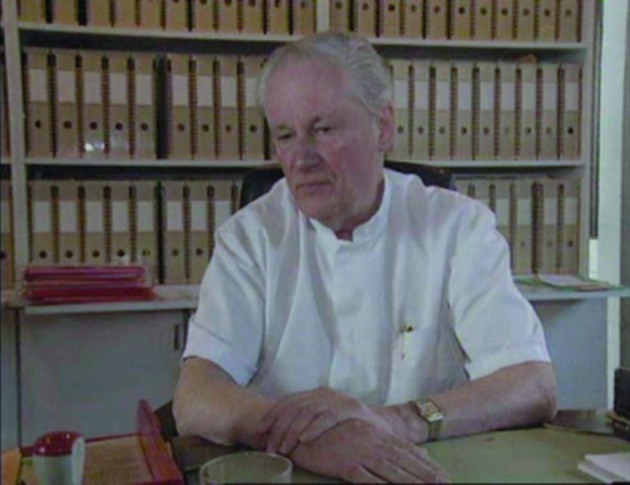


**Fig. 2:**
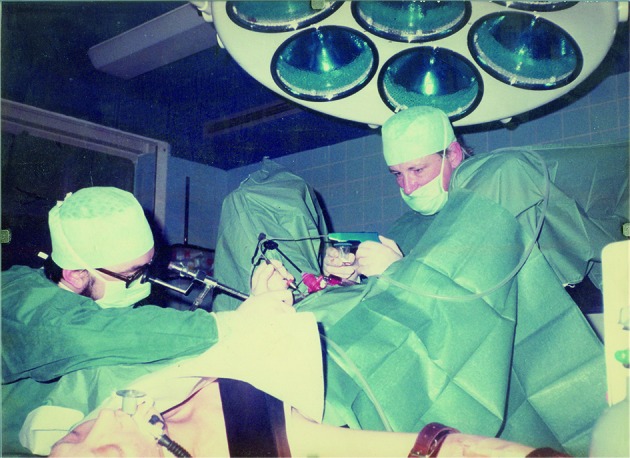
— October 1981, at 3 am during a Saturday night, Jan Gerris (President VVOG) and Bob Schoysman performing a laparoscopic oocyte aspiration in the IVF centre of Vilvoorde.

Robert Schoysman, Bob as we knew him, was born in 1928 in Antwerp, Belgium. He became Professor at the Free University of Brussels and worked for many years in the IVF centre of Vilvoorde.

Between 1954 and 2005 he authored more than 100 PubMed-cited papers and he edited many books of which “Epididymal causes of male infertility: pathogenesis and management” published in 1982 and “Microsurgery of male infertility” published in 1994 were the most popular ones. Bob liked new challenges and innovating techniques, and among the many topics he covered his main interest remained the diagnosis and treatment of male infertility.

The most important topics he pioneered were: How to treat oligozoospermia (1962), surgical procedures for female and male infertility (1967), the exact role of the epididymis in sperm maturation (1987) and the reports and publications on MESA (microsurgical epididymal sperm aspiration), SUZI (subzonal sperm injection) and TESE (testicular sperm extraction).

He was a member of numerous scientific societies all over the world and was the President of the Flemish Society of Obstetrics and Gynaecology (VVOG). He became an honorary member of the VVOG and the Belgian Society of Reproductive Medicine (BSRM).

The work of Bob Schoysman has always been controversial and always ahead of his time on so many issues but he has never shrunk from confronting that controversy. He was a real visionary, obsessed by the challenge of solving the problem of male infertility.

On the occasion of the 2nd meeting of ‘Andrology in the Nineties’ in 1995, I had the privilege to invite three pioneers, Bob Edwards, Howard Jones and Bob Schoysman to Genk, Belgium (Fig. 3, Fig 4). The enthusiasm and the richness of their visionary ideas and beliefs inspired all participants and those who attended the meeting will never forget the fascinating lecture of Bob Schoysman entitled “Plea for realism and honesty in results of infertility treatment’. Immediately after his talk Bob Edwards asked him to write a paper on this topic for the international journal “Human Reproduction” (Schoysman, 1996).

**Fig. 3:**
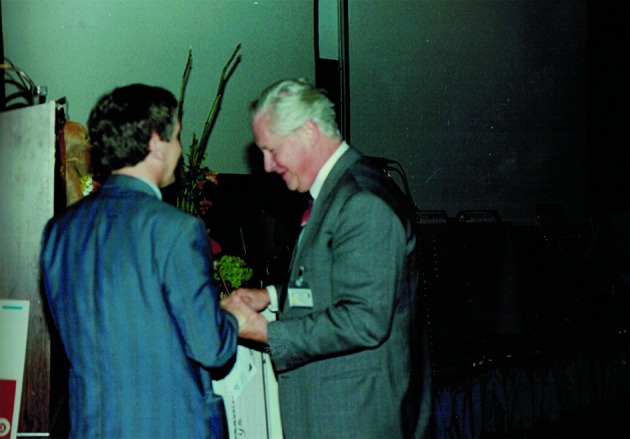
— “Andrology in the Nineties” 1995: Bob Schoysman was awarded by the scientific committee of the meeting.

**Fig. 4:**
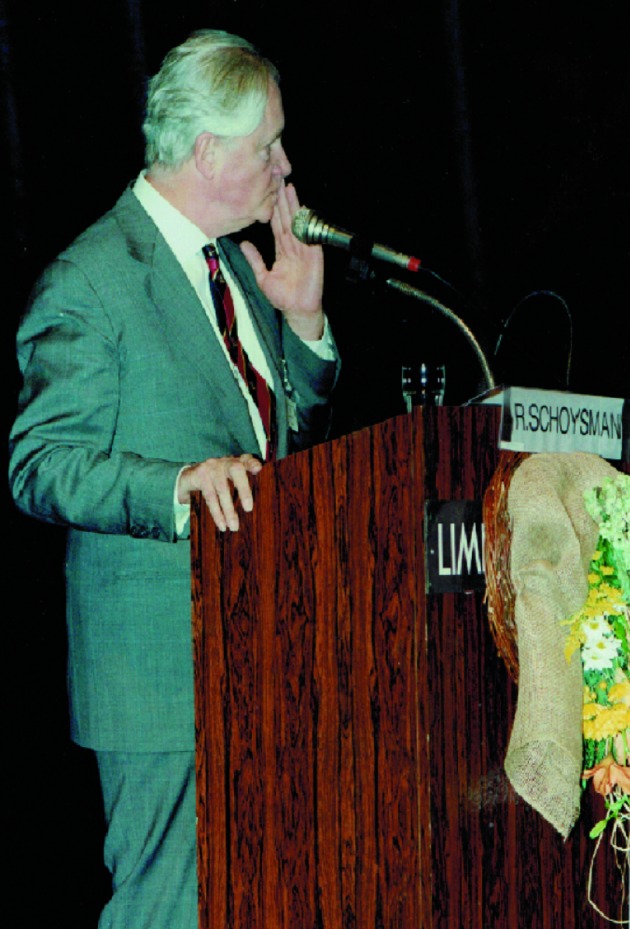
— “Andrology in the Nineties” 1995: Bob Schoysman on stage in Genk giving his famous lecture on ‘A plea for realism and honesty in results of infertility treatment’

“... the solution for each of us is to constantly try to improve the quality of our work and to keep cool and to avoid all grandiloquence. One must not play the game of the escalator. The quality of a medical men’s service must not be left to fantasm... ‘You can fool all the people for a certain time, you can fool some people all the time, but you cannot fool all the people all the time’...”

Bob Schoysman will be remembered internationally as a visionary, a superb teacher and speaker who never failed to fascinate his audience. He had the distinct ability to deliver complex ideas and opinions with impeccable clarity and graceful elegance. As mentor of clinicians and scientists from all over the world he “exported” IVF and male infertility treatment to all corners of the world. Those who worked with him found his inspirational leadership and mentorship as truly grandiose. He convinced and encouraged many people to stay in the discipline of reproductive medicine.

Robert (Bob) Schoysman was a talented physician, a skilled surgeon, an innovative thinker, but above all this, a gentleman and a devoted family man.

**A great man passed away quietly**

Many will continue to remember him, because his whole being loved so many.

A few days ago, he simply called his sweet daughter Anne upon his bed, to say goodbye, as he himself had once bid his lovely wife Andrée, his daughter Françoise and his son Piet farewell.

It was so impressive, the way he decided to let go in all tranquillity, while his hands were yet still searching, and for a moment it seemed like heaven and earth were connected.

Those last days, a playful glance and endearing smile often graced his face, as he was so grateful for the wonderful life that had befallen him. His rise as a physician and as a teacher was inspired by his spouse, Andrée Deboeck, whose fundamental thinking lay at base of a great deal of scientific research. As relentlessly as he had put their collective talents at the service of everything and everyone, so quietly he departed from those people he cherished.

After the dreamy fifties and the roaring sixties, he attempted to reveal to his followers how to direct one’s own mind. With innovatory ideas he showed the value of free and unconstrained thinking, to his colleagues at the VVOG (Flemish Society of Obstetrics and Gynaecology) as well, and it was truly remarkable how he put this valuable talent at the service of his patients and the world. His resilience and science was, first and foremost, fed by Altruism. He had been at the cradle of Andrology and In Vitro Fertilisation, and as a convinced researcher he softly reprimanded all those who didn’t look further than their own horizon; he was proven right.

He understood the art of truly experiencing life and even in the seclusion of those last days, he remained amazed and still searching...

His daughter and son in law, his grandchildren, his nieces and nephews, his friends and fellows, they are left with the riches of a beautiful past, that of a man “like no other”, with a life fulfilled.

In memoriam,


Mireille Merckx
Board member VVOG

